# Circ-0001283 Aggravates Cardiac Hypertrophy by Targeting Myosin Light Chain 3 Protein

**DOI:** 10.34133/research.0626

**Published:** 2025-02-25

**Authors:** Wenjing Wang, Lili Chen, Yiheng Zhao, Shuchen Zhang, Xiang Zhou

**Affiliations:** ^1^ Intensive Care Unit, The Second Affiliated Hospital of Soochow University, Suzhou, China.; ^2^ Central Laboratory, The Second Affiliated Hospital of Soochow University, Suzhou, China.; ^3^ Department of Cardiology, The Second Affiliated Hospital of Nanjing Medical University, Nanjing, China.

## Abstract

Circular RNAs (circRNAs) are differentially expressed in cardiac hypertrophy; however, the exact function and mechanisms during hypertrophy development are still unknown. Here, we explored the role of a newly discovered circRNA in the pathogenesis of myocardial hypertrophy. It was found that circ-0001283 promoted the progression of cardiac hypertrophy by interacting with myosin light chain 3 (MYL3) to inhibit the protein ubiquitination and enhance its protein expression, not by the competitive endogenous RNA mechanism. Further investigation demonstrated that the reduced hypertrophy induced by circ-0001283 knockdown was counteracted by overexpression of MYL3. Mechanistically, MYL3 facilitated myocardial hypertrophy by inducing autophagy in cells via PI3K/Akt/mTOR and ERK signaling pathways. In summary, circ-0001283 can bind directly to MYL3 and up-regulate its expression, thereby promoting autophagy to accelerate cardiac hypertrophy. Circ-0001283 may serve as a potential therapeutic target for cardiac hypertrophy.

## Introduction

Cardiac hypertrophy initially develops as an adaptive response to physiological and pathological stimuli [1]. However, prolonged hypertrophy can switch the heart from a compensated to decompensated state, finally leading to cardiac dysfunction or heart failure [2,3]. Although numerous signaling pathways involved in the mechanisms of cardiac hypertrophy have been identified, effective therapeutic targets are still lacking.

In recent decades, noncoding RNA has gradually become a major focus of research in many biological processes of cardiovascular diseases. Circular RNA (circRNA) is a recently identified class of noncoding RNA, originating from the back-splicing of precursor mRNA, and is ubiquitously present in eukaryotic cells [4]. Existing studies have shown that some circRNAs contain microRNA (miRNA) binding sites, enabling them to function effectively as miRNA sponges in cells, thus regulating the expression of genes implicated in myocardial hypertrophy by targeting specific miRNAs [5]. Argonaute 2 (AGO2) is an essential component of this sponge mechanism [6]. In addition, circRNAs can interact with transcription factors or proteins, affecting their stability and function, thus contributing to the pathogenesis of cardiac hypertrophy [7]. These findings indicate that further investigation into circRNAs could substantially advance the understanding of molecular mechanisms underlying pathological cardiac hypertrophy.

Collectively, we established a mouse model of cardiac hypertrophy through transverse aortic constriction (TAC) and identified a novel circRNA, circ-0001283, which was markedly up-regulated in the hearts of hypertrophic mice, as revealed by circRNA sequencing (circRNA-seq) analysis. Using RNA–protein pull-down assays and mass spectrometry, we discovered that circ-0001283 directly interacted with the myosin light chain 3 (MYL3) protein. We further explored the potential mechanisms by which these interactions may affect the progression of cardiac hypertrophy, with a particular focus on the relevant downstream signaling pathways.

## Results

### Circ-0001283 expression was up-regulated during TAC-induced cardiac hypertrophy in mice

**Fig. 1.**
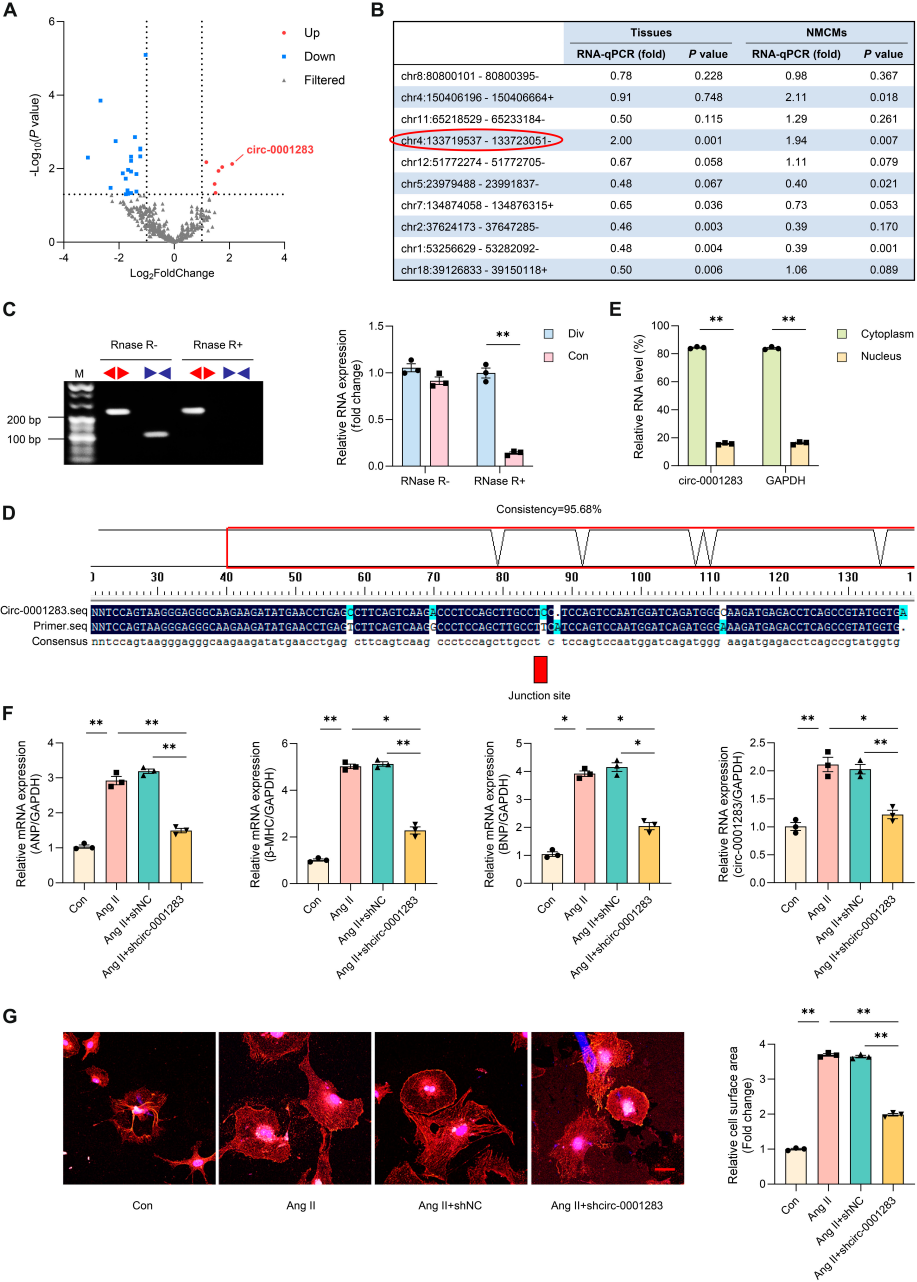
Knockdown of circ-0001283 relieved neonatal mouse cardiomyocyte (NMCM) hypertrophy. (A) Volcano plot showing differentially expressed circRNAs in transverse aortic constriction (TAC) and control mice. (B) Differences in RNA expression levels in heart tissues of TAC and control mice (left) and in NMCMs (right) treated with or without angiotensin II (Ang II; 1 μg/ml), measured by quantitative real-time PCR (qRT-PCR). (C) PCR was performed to determine circRNAs and linear RNAs in NMCMs treated with RNase R. Convergent (Con; blue) and divergent (Div; red) primers were utilized to amplify linear and back-splicing products. (D) RNA sequencing to identify junction site of circ-0001283. (E) qRT-PCR analysis of circ-0001283 expression in cytoplasm and nuclear fractions of NMCMs. (F) mRNA expression of cardiac hypertrophy markers atrial natriuretic factor (ANP), myosin heavy chain-β (β-MHC), B-type natriuretic peptide (BNP), and circ-0001283 level in cells stimulated by Ang II (1 μg/ml), with or without shcirc-0001283. (G) Left: Effect of circ-0001283 knockdown on cell cross-sectional area, assessed by measuring sarcomere organization. Scale bar, 20 μm. Right: Quantitative analysis of cell surface area. *P < 0.05, **P < 0.01, *n* = 3.

Differences in circRNAs in mouse hearts subjected to TAC procedure were evaluated compared to control mice using circRNA-seq assays. Among the 4,510 identified circRNAs, 31 exhibited differential expression, comprising 25 down-regulated and 6 up-regulated circRNAs (Fig. 1A). We selected 10 circRNAs with high confidence and significant differential expression for further verification by quantitative real-time polymerase chain reaction (qRT-PCR). Of these 10 mouse circRNAs, 9 homologous circRNAs were found in humans (Table S1). Two circRNAs (chr4:133719537-133723051- and chr1: 53256629-53282092-) were ultimately confirmed to be differentially expressed in both mouse hearts and neonatal mouse cardiomyocytes (NMCMs) (Fig. 1B). Notably, circRNA chr4:133719537-133723051- termed circ-0001283 (homologous to has-circ-0008494 in humans) was found to be up-regulated and selected for further investigation. Circ-0001283 is located on chromosome 4 in the mouse genome Arid1a, with its sequence detailed in Table S2. To verify the loop structure of circ-0001283, specific convergent and divergent primers were used to amplify the canonical or back-spliced forms of Arid1a. qRT-PCR using the divergent primers indicated that circ-0001283 was not affected by RNase-R (an exoribonuclease that digests linear RNAs) digestion, while the PCR products from linear Arid1a mRNA amplified by the convergent primers disappeared after RNase-R digestion (Fig. 1C). Sanger sequencing confirmed the circular structure of circ-0001283 (Fig. 1D).

### Knockdown of circ-0001283 ameliorated NMCM hypertrophy

We investigated the regulatory role of circ-0001283 in NMCM hypertrophy induced by angiotensin II (Ang II). Expression levels of circ-0001283 were higher in the cytoplasm of NMCMs than in the nucleus (Fig. 1E). Additionally, mRNA levels of the myocardial hypertrophy indicators atrial natriuretic factor (ANP), myosin heavy chain-β (β-MHC), and B-type natriuretic peptide (BNP) were increased in cells following stimulation with Ang II (Fig. 1F). The knockdown of circ-0001283 effectively mitigated Ang II-induced hypertrophy, as evidenced by a reduction in NMCM surface areas (Fig. 1G) and decreased mRNA expression levels of ANP, β-MHC, and BNP (Fig. 1F). These results indicated that circ-0001283 knockdown inhibited Ang II-induced NMCM hypertrophy.

### Circ-0001283 promoted MYL3 protein expression to regulate NMCM hypertrophy

**Fig. 2.**
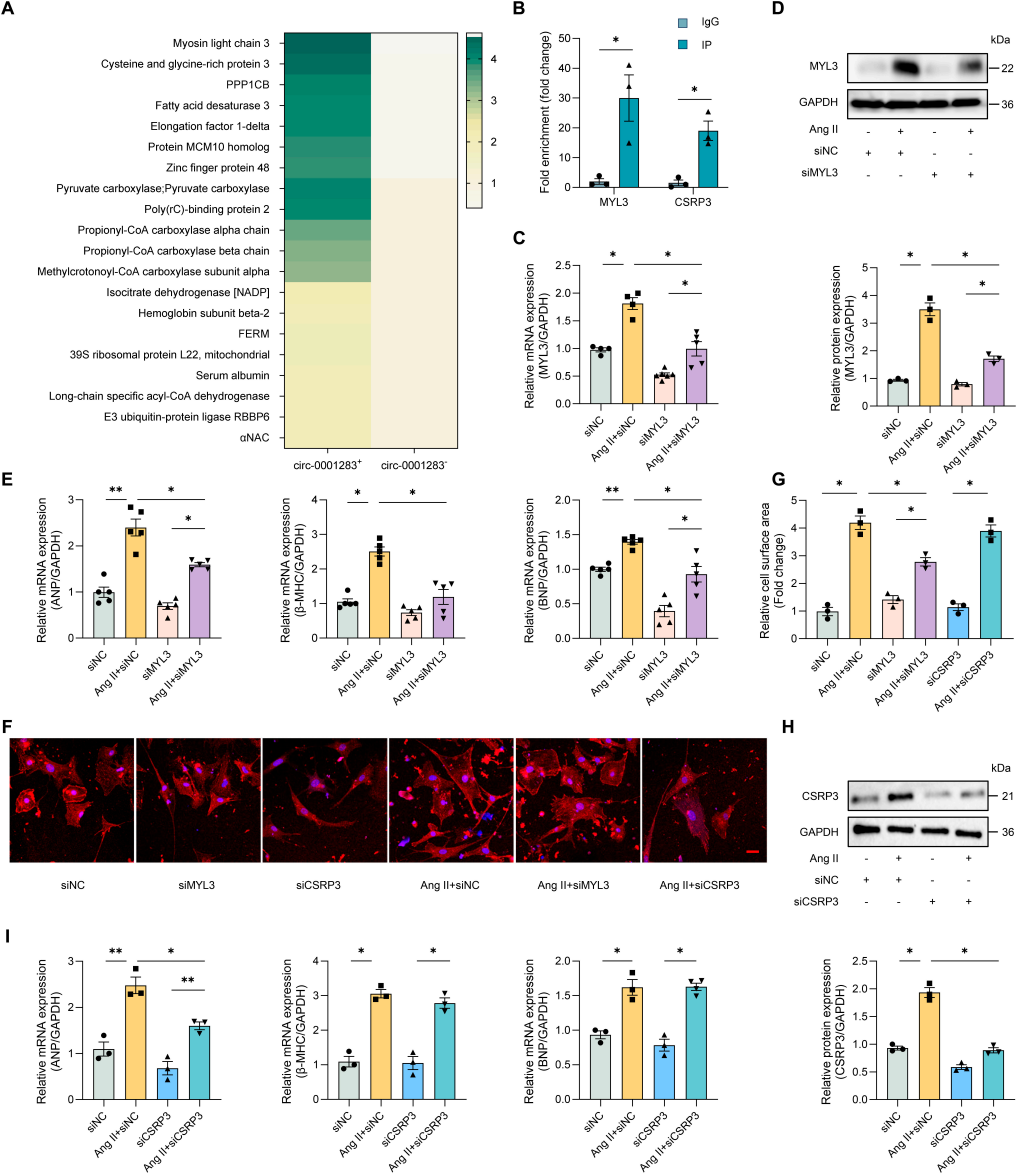
Knockdown of myosin light chain 3 (MYL3) alleviated NMCM hypertrophy. (A) Hierarchical cluster analysis of 20 proteins differentially expressed in circ-0001283-positive and circ-0001283-negative groups, distinguished by circ-0001283 RNA pull-down. (B) RNA immunoprecipitation assay (RIP) showing direct binding of circ-0001283 to MYL3 or cysteine and glycine-rich protein 3 (CSRP3). (C) qRT-PCR analysis of MYL3 mRNA expression in NMCMs with or without siMYL3 and Ang II treatment (1 μg/ml) (*n* = 5). (D) Western blotting of MYL3 expression in NMCMs with or without siMYL3 and Ang II treatment (1 μg/ml). (E) qRT-PCR analysis of ANP, β-MHC, and BNP mRNA expression levels in cells with or without siMYL3 and Ang II treatment (1 μg/ml) (*n* = 5). (F) NMCM hypertrophy assessed by measuring sarcomere organization. Scale bar, 20 μm. (G) Quantification of cell surface area. (H) Western blotting of CSRP3 expression in cells with or without siCSRP3 and Ang II treatment (1 μg/ml). (I) qRT-PCR analysis of ANP, β-MHC, and BNP mRNA expression levels in cells with or without siCSRP3 and Ang II treatment (1 μg/ml). *P < 0.05, **P < 0.01, *n* = 3.

We further explored the molecular mechanisms by which circ-0001283 regulated NMCM hypertrophy. An RNA–protein pull-down assay was operated in NMCMs using biotinylated circ-0001283. The proteins extracted from the pull-down materials were purified to determine which proteins bound specifically to biotinylated circ-0001283 using mass spectrometry. Some proteins were specifically enriched in the circ-0001283-positive group, with the exception of AGO2, an indicator protein known to function as a sponge for circRNAs (Fig. 2A). We selected 2 proteins, MYL3 and cysteine and glycine-rich protein 3 (CSRP3), which are abundantly expressed in hearts and are closely associated with cardiovascular diseases, for further investigation. These proteins are involved in the regulation of myocardial contraction and hypertrophy through diverse cellular signaling pathways [8–11]. RNA immunoprecipitation (RIP) analysis of RNA-binding MYL3 or CSRP3 (Fig. 2B) demonstrated that both MYL3 and CSRP3 precipitated significantly more circ-0001283 compared to immunoglobulin G (IgG), indicating that MYL3 and CSRP3 directly bind to circ-0001283.

We hypothesized that circ-0001283 regulated cardiac hypertrophy via MYL3 or CSRP3. MYL3 mRNA (Fig. 2C) and protein (Fig. 2D) expression levels were up-regulated in NMCMs treated with Ang II and decreased again in siMYL3-treated cells. The expression levels of the hypertrophy indicators ANP, β-MHC, and BNP, and cell surface areas were all increased in Ang II-treated NMCMs. These parameters were subsequently reduced after the knockdown of MYL3 (Fig. 2E to G). We also assessed the effect of CSRP3 expression on NMCM hypertrophy, analogous to the approach taken with MYL3. CSRP3 was elevated in Ang II-stimulated cells (Fig. 2H), but CSRP3 knockdown failed to reduce the hypertrophy indices and cell surface area (Fig. 2F and I).

**Fig. 3.**
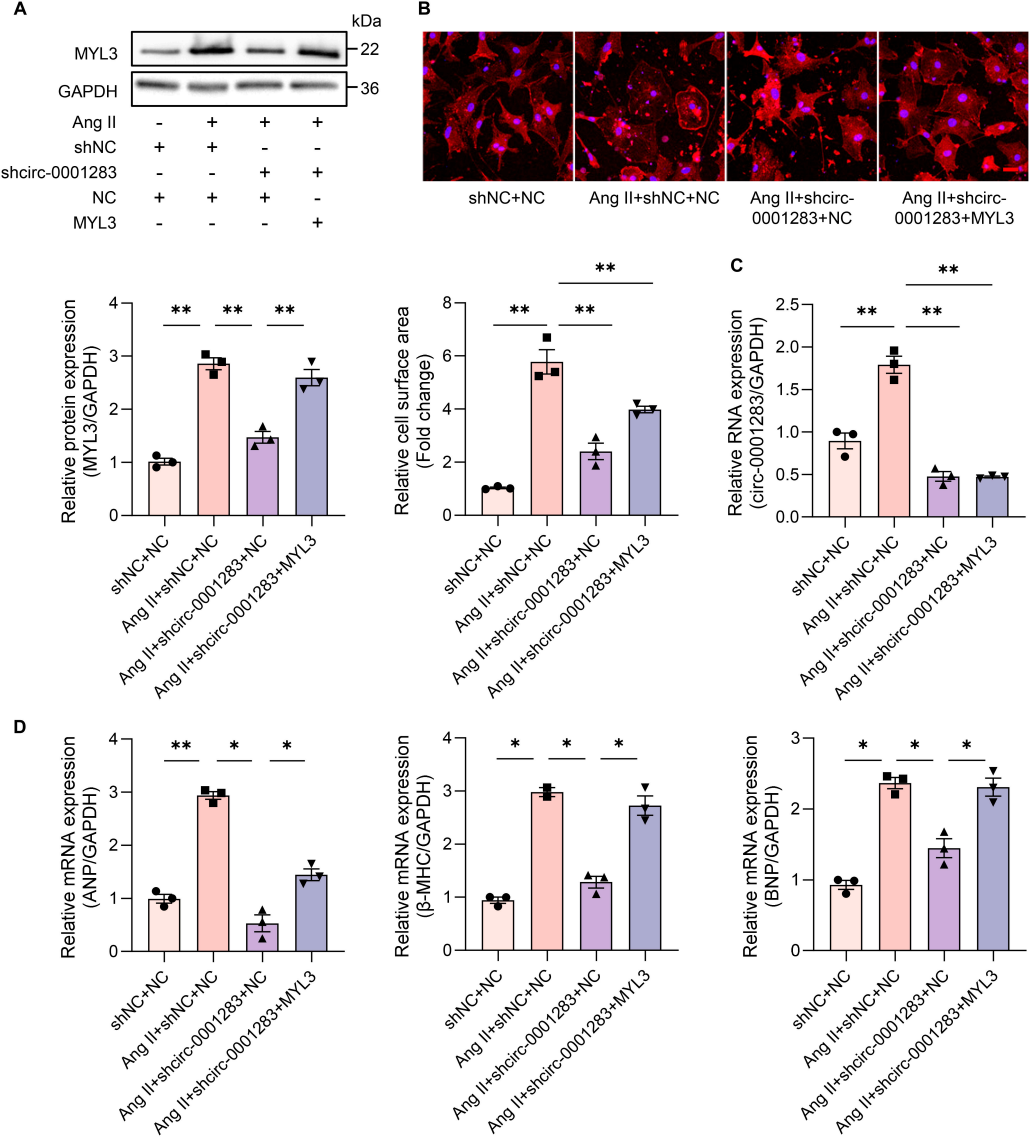
Circ-0001283 regulated NMCM hypertrophy mainly via MYL3. (A) Western blot of MYL3 expression in NMCMs exposed to Ang II (1 μg/ml) and transfected with shcirc-0001283 and/or MYL3. (B) Top: Hypertrophy assessed by measuring sarcomere organization. Scale bar, 20 μm. Bottom: Quantitative analysis of cell surface area. qRT-PCR analysis of circ-0001283 (C), ANP, β-MHC, and BNP (D) expression levels in NMCMs exposed to Ang II (1 μg/ml) and transfected with shcirc-0001283 and/or MYL3. *P < 0.05, **P < 0.01, *n* = 3.

Furthermore, knockdown of circ-0001283 mitigated NMCM hypertrophy, which was aggravated by MYL3 overexpression. Consequently, circ-0001283 exacerbated Ang II-induced NMCM hypertrophy through the regulation of MYL3 (Fig. 3A to D). However, CSRP3 overexpression had no such effect (Fig. S1).

### Circ-0001283 inhibited proteasome degradation to promote MYL3 protein expression

**Fig. 4.**
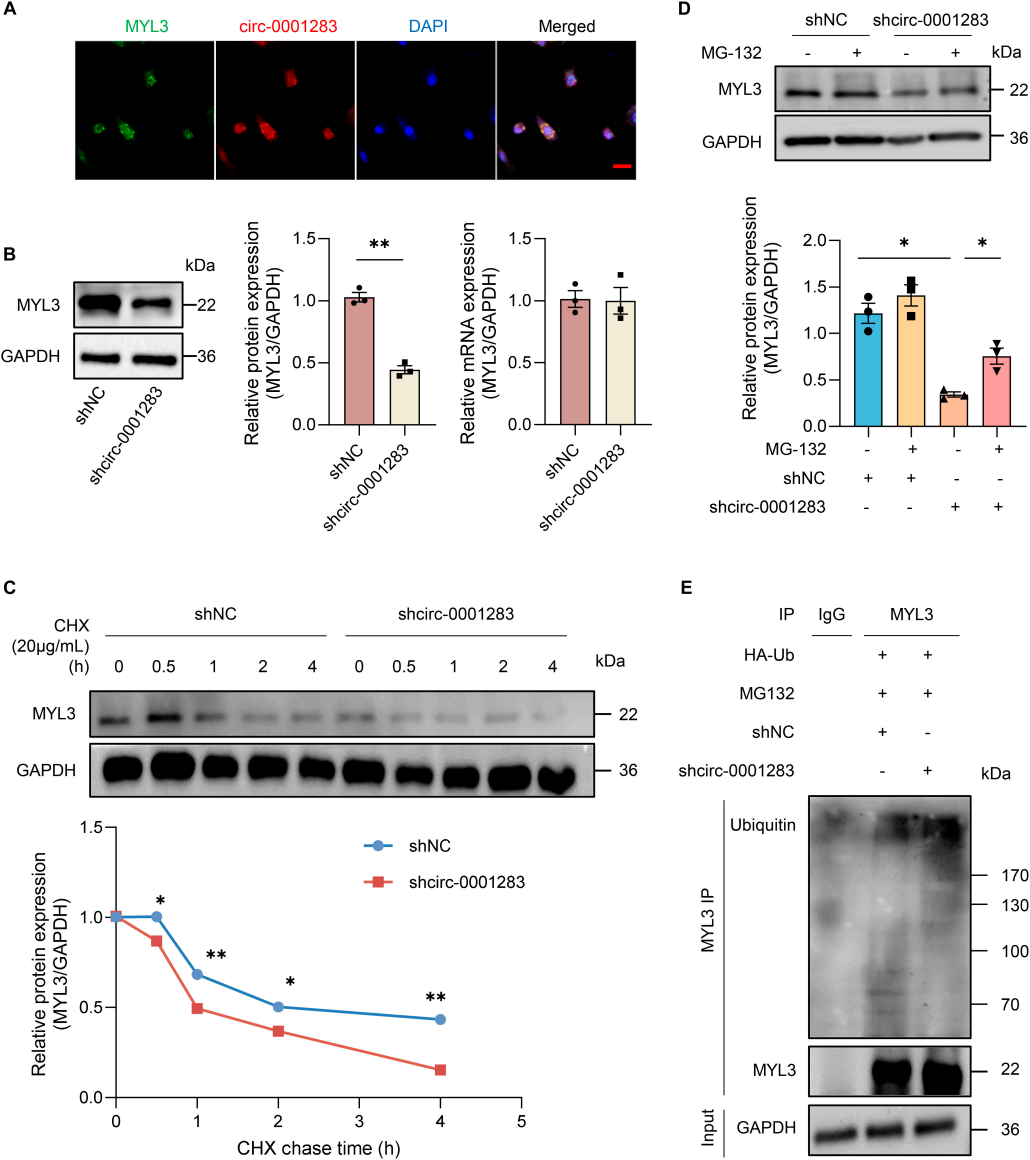
Circ-0001283 prevented MYL3 proteasomal degradation to regulate its protein expression in NMCMs. (A) Colocalization of circ-0001283 and MYL3 in NMCMs by immunofluorescence–fluorescence in situ hybridization (IF-FISH). Scale bar, 20 μm. (B) Western blot (left, middle) and qRT-PCR (right) analyses of MYL3 expression in NMCMs exposed to Ang II (1 μg/ml). (C) Western blot of MYL3 protein levels in cells with or without circ-0001283 knockdown measured after treatment with cycloheximide (CHX; 20 μg/ml) at 0, 0.5, 1, 2, and 4 h. (D) Western blot of MYL3 protein levels in cells with or without circ-0001283 knockdown measured after treatment with MG-132 (20 μM) for 2 h. (E) NMCMs transfected with ubiquitin (Ub) and shcirc-0001283 after treatment with MG-132 were subjected to immunoprecipitation assay with MYL3 antibody, and Western blot was performed using ubiquitin antibody. *P < 0.05, **P < 0.01, *n* = 3.

Immunofluorescence–fluorescence in situ hybridization (IF-FISH) showed that MYL3 and circ-0001283 were colocated in NMCMs (Fig. 4A). Western blot assays revealed that knockdown of circ-0001283 reduced MYL3 protein but not mRNA expression (Fig. 4B). These findings suggested that circ-0001283 might facilitate MYL3 protein expression through protein degradation. Cycloheximide (CHX) assay revealed that the half-life of MYL3 protein was shorter in the circ-0001283 knockdown compared with the control group (Fig. 4C). Moreover, we used the proteasome inhibitor MG-132 to investigate if circ-0001283 affected MYL3 protein stability through proteasome. MG-132 rescued the reduction of MYL3 caused by circ-0001283 inhibition, indicating that circ-0001283 reduced MYL3 protein degradation in NMCMs (Fig. 4D). Furthermore, we performed a coimmunoprecipitation assay in NMCMs cotransfected with shcirc-0001283 virus and ubiquitin plasmids to determine if circ-0001283 inhibited MYL3 protein degradation via a ubiquitination-dependent mechanism. Circ-0001283 knockdown significantly increased the levels of ubiquitinated MYL3 protein, which accelerated MYL3 proteasomal degradation (Fig. 4E). These results showed that circ-0001283 prevented MYL3 proteasomal degradation, thereby regulating its protein expression through a direct interaction between the circRNA and protein.

### MYL3 aggravated NMCM hypertrophy by inducing autophagy via PI3K/Akt/mTOR and ERK signaling pathways

**Fig. 5.**
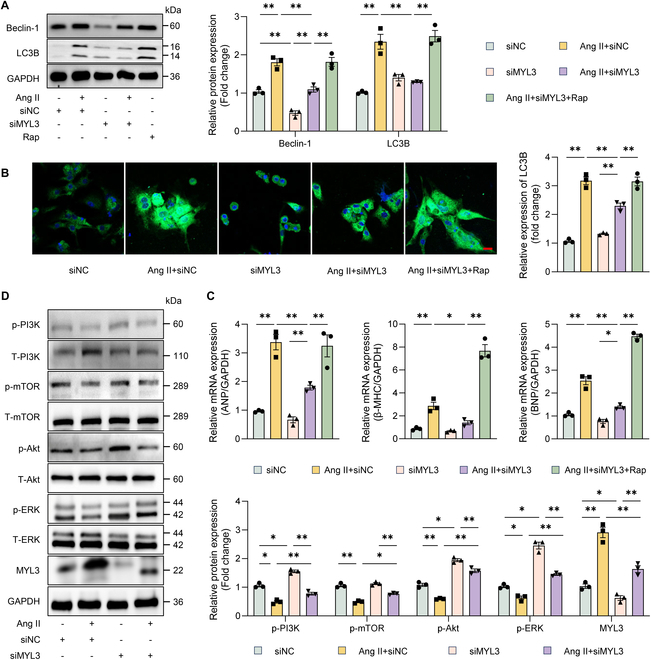
MYL3 regulated PI3K/Akt/mTOR and ERK pathways in NMCMs. (A) Western blot showing expression levels of Beclin-1, microtubule-associated protein 1 light chain 3 β (LC3B), and MYL3 in NMCMs transfected with siMYL3 with or without Ang II (1 μg/ml) and rapamycin (Rap; 100 nM). LC3B (14 kDa)/GAPDH was used to calculate LC3B expression. (B) Expression of LC3B in NMCMs detected by IF. Scale bar, 20 μm. (C) qRT-PCR analysis of ANP, β-MHC, and BNP expression levels in cells transfected with siMYL3 with or without Ang II (1 μg/ml) and Rap (100 nM). (D) Western blot showing expression of phospho (p)-phosphoinositide 3-kinase (PI3K), p-mammalian target of rapamycin (mTOR), p-Akt, p-p44/42 mitogen-activated protein kinase (MAPK, ERK1/2), and MYL3 in cells transfected with siMYL3 with or without Ang II (1 μg/ml) and Rap (100 nM). Scale bar, 10 μm. *P < 0.05, **P < 0.01, *n* = 3.

MYL3 is a member of the myosin family, and some of its members are associated with autophagy, especially the phosphoinositide 3-kinase (PI3K)/Akt/mammalian target of rapamycin (mTOR) and p44/42 mitogen-activated protein kinase (MAPK, ERK) signaling pathways according to previous researches [12–15]. We thus proposed that MYL3 might also contribute to the process of autophagy during NMCM hypertrophy. It was found that Ang II increased protein expression of the autophagy markers Beclin-1 and microtubule-associated protein 1 light chain 3 β (LC3B), and knockdown of MYL3 decreased the up-regulation stimulated by Ang II (Fig. 5A). However, the inhibition of autophagy was relieved in cells treated with rapamycin (Rap), as evidenced by the increased expression of Beclin-1 and LC3B proteins after Rap stimulation in hypertrophic NMCMs transfected with siMYL3 (Fig. 5A and B). This observation suggested that autophagic process could be reactivated by Rap. In addition, knockdown of MYL3 resulted in the suppression of hypertrophy markers such as ANP, BNP, and β-MHC in hypertrophic cells, which was again aggravated in the presence of Rap (Fig. 5C). Furthermore, PI3K/Akt/mTOR and ERK signaling pathways were implicated in the regulation of MYL3-mediated hypertrophy. Western blot analyses demonstrated that Ang II inhibited the phosphorylation levels of PI3K/Akt/mTOR and ERK while concurrently increasing MYL3 protein expression. Conversely, siMYL3 transfection in NMCMs activated the PI3K/Akt/mTOR and ERK pathways (Fig. 5D).

Given that circ-0001283 regulated NMCM hypertrophy through the modulation of MYL3 expression, we determined the role of circ-0001283 in influencing autophagy and the PI3K/Akt/mTOR pathway. As expected, circ-0001283 knockdown in hypertrophic NMCMs led to the reactivation of phosphorylation levels of PI3K/Akt/mTOR, without affecting ERK phosphorylation. However, phosphorylation levels, including that of ERK, were subsequently reduced following MYL3 overexpression. In alignment with these findings, circ-0001283 knockdown suppressed the protein expression of Beclin-1 and LC3B, both of which were elevated in MYL3-overexpressing hypertrophic NMCMs (Fig. S2). Overall, the up-regulation of circ-0001283 induced by Ang II activated autophagy, thereby exacerbating hypertrophy through the promotion of MYL3 expression. This process involved the PI3K/Akt/mTOR and ERK signaling pathways.

### Circ-0001283 facilitated cardiac hypertrophy by regulating MYL3 expression in mice

**Fig. 6.**
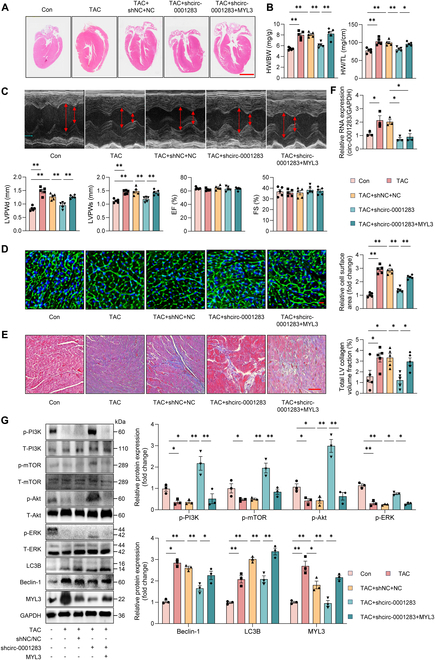
Circ-0001283 aggravated cardiac hypertrophy by regulating MYL3 expression in mice. Mice were injected with AAV9-shcirc-0001283 or AAV9-shNC (5 × 10^10^ vector genomes per mouse) via myocardial injection to knock down expression of circ-0001283, and ADV-MYL3 or ADV-NC (5 × 10^8^ vector genomes per mouse) was used to achieve overexpression of MYL3. (A) Representative hematoxylin and eosin (H&E)-stained longitudinal sections of hearts. Scale bar, 2 mm. (B) Heart weight/body weight ratio (HW/BW) and heart weight/tibia length (HW/TL) were calculated. (C) Top: Representative echocardiography images of mice 4 weeks after TAC. Bottom: Left ventricular posterior wall thickness at end-diastole (LVPWd), left ventricular posterior wall thickness at end-systole (LVPWs), ejection fraction (EF), and fractional shorting (FS) were measured by echocardiography 4 weeks after TAC. (D) Representative wheat germ agglutinin (WGA)-stained left ventricular muscle sections and quantitative analysis. Scale bar, 100 μm. (E) Representative Masson’s trichrome-stained histological sections and quantitative analysis. Scale bar, 100 μm. (F) qRT-PCR showing expression of circ-0001283 in mice (*n* = 3). (G) Western blot analysis of expression levels of p-PI3K, p-mTOR, p-Akt, p-ERK, Beclin-1, LC3B, and MYL3 in heart tissues (*n* = 3). *P < 0.05, **P < 0.01, *n* = 5.

We evaluated the functional roles of circ-0001283 and MYL3 during pathological myocardial hypertrophy using an in vivo model. TAC mice exhibited pronounced myocardial hypertrophy and fibrosis, which were reversed by the knockdown of circ-0001283. This was evidenced by reductions in heart-to-body weight ratio, heart weight-to-tibia length ratio, ventricular wall thickness, relative cell area, and left ventricular collagen volume. In addition, MYL3 overexpression exacerbated cardiac hypertrophy in TAC mice with circ-0001283 knockdown (Fig. 6A to E). Furthermore, circ-0001283 regulated autophagy and inhibited the PI3K/Akt/mTOR and ERK signaling pathways, potentially contributing to the pathogenesis of myocardial hypertrophy (Fig. 6F and G). Moreover, the knockdown of either circ-0001283 or MYL3 alone in TAC mice resulted in reduced myocardial hypertrophy compared to the TAC + NC group (Fig. S3).

## Discussion

**Fig. 7.**
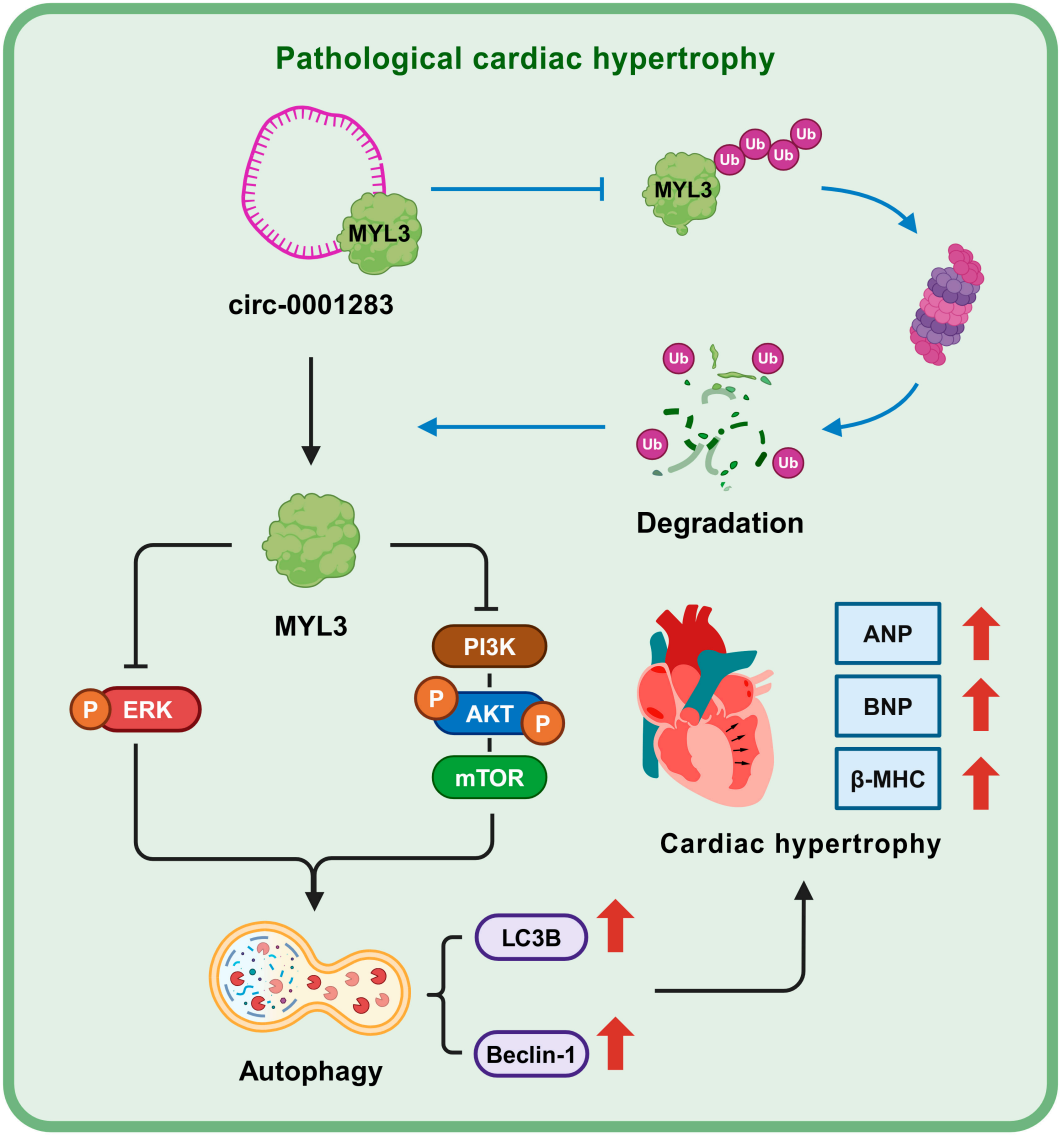
The circ-0001283 is involved in the pathogenesis of cardiac hypertrophy.

Our current study demonstrated that the novel circRNA, circ-0001283, was up-regulated during cardiac hypertrophy and interacted with MYL3 protein in the cytoplasm. Circ-0001283 decreased the ubiquitylation of MYL3, thereby inhibiting the proteasomal degradation and enhancing its expression levels. The increased expression of MYL3 promoted autophagy and exacerbated cardiac hypertrophy through the modulation of PI3K/Akt/mTOR and ERK signaling pathways. These findings indicated that circ-0001283 could serve as a promising therapeutic target in cardiac hypertrophy (Fig. 7).

To date, research on circRNAs related to cardiac hypertrophy remains sparse. Several studies have elucidated the regulatory functions of circRNAs in cardiac hypertrophy, with a primary focus on the miRNA-mediated sponging mechanism that may affect the expression of downstream target genes [16,17]. Direct evidence for circRNA–protein interactions is still insufficient. Some investigations have shown that circRNAs can participate in RNA-binding protein mechanisms within the nucleus, regulating gene expressions by interactions with transcription factors [18,19]. However, our study discovered that circ-0001283 was predominantly localized in the cytoplasm of cardiomyocytes and was up-regulated during myocardial hypertrophy. It facilitated the recruitment of MYL3, inhibiting its degradation via the ubiquitin–proteasome pathway, thus augmenting MYL3 expression. These findings filled the gap in knowledge about the interactions between circRNAs and proteins, indicating that circRNAs may represent new avenues for the posttranslational regulation of protein expression in the context of cardiac hypertrophy.

MYL3 is a constituent of the basic myosin light chain family, which includes MYL1, MYL3, and MYL4, and is distinguished by a unique N-terminal domain and actin-binding capabilities that are crucial for the establishment of robust myosin cross-links [20,21]. Consequently, MYL3 is widely acknowledged as a molecular “motor” of the cytoskeleton [22]. Research on the role of MYL3 in the diagnosis and treatment of hypertrophic cardiomyopathy has predominantly concentrated on genetic variants, with limited information available regarding the function of MYL3 itself in the regulation of cardiac hypertrophy. Our study demonstrated that the down-regulation of MYL3 expression can effectively alleviate pathological cardiac hypertrophy, thereby offering new insights into the role of MYL3 in cardiac hypertrophy.

Previous studies have demonstrated that members of the myosin family play a crucial role in the regulation of cardiomyocytes, particularly in the context of autophagy [23]. In light of this, we conducted an in-depth investigation into the role of MYL3, a specific member of the myosin family, in the autophagic processes of NMCMs. Autophagy is also recognized as a pro-death factor in myocardial hypertrophy [24]. Ang II induced autophagy, thereby facilitating the development of cardiac hypertrophy through the augmentation of cellular dysfunction and apoptosis [25–27]. Dai et al. [28,29] emphasized that Ang II treatment significantly increases the LC-3 II/I ratio in myocardial tissues. mTOR, a key upstream regulatory factor for autophagy induction, has been extensively studied, particularly in relation to the PI3K/Akt/mTOR and ERK signaling pathways [30]. In this study, MYL3 was found to regulate the PI3K/Akt/mTOR and ERK signaling pathways, thereby promoting autophagy and aggravating myocardial hypertrophy. These results indicated that inhibiting MYL3 expression via circ-0001283 and reducing autophagy may represent a promising treatment approach for cardiac hypertrophy.

In conclusion, our study identified a novel circRNA, circ-0001283, which exacerbated cardiac hypertrophy in mice by targeting MYL3 and activating autophagy. These findings underscore the importance of circ-0001283 and present its promising application as a therapeutic molecule for mitigating the progression of cardiac hypertrophy.

## Materials and Methods

### The isolation of NMCMs

NMCMs were dissociated from the hearts of newborn mice [1 or 2 d old, Shanghai Laboratory Animals Center (SLAC), China], as described below. First, the mouse hearts were exposed after thoracotomies were performed under anesthesia with pentobarbital (200 mg/kg) through intraperitoneal administration. The heart was removed and separated from adipose tissues and blood vessels in Dulbecco’s modified Eagle’s medium (DMEM). Tiny pieces were obtained by shearing the hearts, following digestion in 0.125% trypsin (Beyotime, Shanghai, China) for 10 min at 37 °C. The cell suspensions were separated, redigested in 0.05% collagenase (Sigma-Aldrich, St Louis, MO, USA) for 30 min at 37 °C, and supplemented with isometric DMEM containing 10% fetal bovine serum (FBS; Gibco) to stop the digestion. The cell suspensions were then centrifuged at 1,000*g* for 5 min, washing the cell pellets twice using phosphate-buffered saline (PBS) after discarding the supernatants, then resuspending cells in DMEM containing 10% FBS before preplating for 1 h to detach fibroblasts. Non-adherent NMCMs in DMEM with 10% FBS and 1% 5-bromodeoxyuridine were seeded on 6-well plates at a density of 1 × 10^6^ cells per well in a humid environment of 95% air/5% CO_2_ at 37 °C. After 48 h in culture, NMCMs were processed as required. NMCM hypertrophy was induced by incubation with 1 μg/ml Ang II for 24 h. Rap (MedChemExpress, NJ, USA) (100 nM) was used to activate cell autophagy for 24 h.

### Animal model and echocardiography

The Ethics Committee of Soochow University granted approval for the animal experiments in this study. TAC was conducted to construct the cardiac hypertrophy model, as described previously [31]. Briefly, 8- to 10-week-old male C57BL/6 mice (SLAC) were randomly assigned to the control or TAC group. The mice were anesthetized by intraperitoneal administration of a mixed anesthetic cocktail containing ketamine and xylazine (100 and 2.5 mg/kg, respectively). The arcus aortae were visualized via left anterior thoracotomy. A TAC model was established by tying a 6-0 stitch near the aorta and padding a 27-gauge needle into the arched area between the left and right carotid arteries. The needle was then quickly withdrawn, and the wound was closed using a 4-0 suture. The same procedure was conducted for mice in the control group, except that the suture was not tied. Following surgery, the mice were placed on a thermostatic blanket and observed until full recovery before proceeding to the next step.

Adeno-associated virus serotype 9 (AAV9) vectors were served to knock down expression of circ-0001283, and adenovirus vector (ADV) was used to achieve overexpression of MYL3 in the animal models. The sequence fragments for circ-0001283 and MYL3 were obtained by PCR and cloned into vector AAV-GP-11 (pAAV-MCS) or pDKD-U6-CMV-mCherry to extract the recombinant plasmid. The corresponding recombinant AAV9 or ADV vectors were then generated through transient transfection in HEK293 cells. AAV9-shcirc-0001283 and ADV-MYL3, together with their respective noncoding control vectors (AAV9-shNC and ADV-NC), were obtained from GenePharma (Shanghai, China). Mice were injected with AAV9-shcirc-0001283 or AAV9-shNC (5 × 10^10^ vector genomes per mouse, *n* = 8 mice per group) via myocardial injection while TAC surgery was conducted. ADV-MYL3 or ADV-NC (5 × 10^8^ vector genomes per mouse) was injected 7 d after TAC surgery via myocardial injection. Briefly, the virus was quickly injected at 3 to 5 points with a microinjector at the thicker part of the myocardium; the operator then gently replaced the heart in the thoracic cavity, squeezed it to expel the air, and tightened the ligation incision.

Mice underwent M-mode echocardiography 21 d after the second injection, using a Vevo 2100 imaging system (Fujifilm VisualSonics, Toronto, Canada). Mice were operated under anesthesia by inhalation of 2% isoflurane. The probe was placed on the left sternum to identify the horizontal mitral valve in left ventricular long-axis view. Left ventricular posterior wall thickness at end-diastole (LVPWd), left ventricular posterior wall thickness at end-systole (LVPWs), left ventricular ejection fraction (LVEF), and left ventricular fractional shortening (LVFS) were then determined. The measurements were averaged over 3 consecutive cardiac cycles by an experienced technician. The mice were then sacrificed by cervical dislocation after the intraperitoneal injection of pentobarbital (200 mg/kg), then removing rapidly the heart to weigh them and perform other measurements.

For circ-0001283 or MYL3 knockdown alone, AAV9-shcirc-0001283, AAV9-shMYL3, or AAV9-shNC was intravenously injected into mice 3 d after TAC. Similarly, the hearts were stripped off and used for other measurements as described above.

### Morphological and histological analyses

Mouse hearts were immediately immersed in 4% paraformaldehyde (pH 7.4) for 12 h. After dehydration with ethanol, paraffin was used to embed the specimens for subsequent procedures. Histological examinations were performed by staining 5-μm-thick sections using hematoxylin and eosin (H&E). Fluorescein isothiocyanate (FITC)-conjugated wheat germ agglutinin (WGA; Sigma-Aldrich) staining was used to determine cell cross-sectional areas. Cardiac fibrosis was evaluated by staining the heart sections with standard Masson’s trichrome (Sigma-Aldrich) following the manufacturer’s instructions.

### The circRNA-seq

Total RNA samples of mouse hearts were extracted utilizing TRIzol reagent (Invitrogen, Carlsbad, CA, USA). After processing, total RNA quantification was conducted on the NanoDrop ND-1000 spectrophotometer (Thermo Fisher, Wilmington, DE, USA). Briefly, a circRNA enrichment kit (Cloudseq Inc., Shanghai, China) was used to preprocess 5 μg of total RNA to obtain circRNA. Next, pretreated RNAs were built into a circRNA-seq library with the Truseq Stranded Total RNA library prep kit (Illumina, San Diego, CA, USA) according to the manufacturer’s instructions. Quality and quantity assurance analysis of the libraries was performed on the Bioanalyzer 2100 system (Agilent Technologies, Santa Clara, CA, USA). The libraries were chemically fragmented into single-stranded RNA molecules, captured on Illumina flow cells, amplified into clusters in situ, and finally sequenced for 150 cycles using an Illumina HiSeq sequencer, in accordance with the manufacturer’s protocol. The sequencing process was performed by Cloudseq Inc., and qualitative visualization of circRNA-seq reads was carried out with the Illumina HiSeq 4,000 sequencer. After trimming the 3′-adaptor and removing low-quality reads with Cutadapt software (v1.9.3), the reference genome/transcriptome was used to align the reads using STAR software. The detection and annotation of circRNAs were conducted utilizing DCC software, and the confirmed circRNAs were noted using the circBase and circ2Trait disease databases. Normalization of primordial junction reads for each sample was based on total read number and subjected to log_2_ transformation. The *t* test was performed to differentiate and discriminate the discrepantly expressed circRNAs. The criteria for discrepantly expressed genes were defined as a *P* value of ≤0.05 and an absolute fold change value of ≥2, followed by qPCR validation and structural verification for selected target circRNAs to determine their expression patterns. Given the limited number of differentially expressed circRNAs, false discovery rate (FDR) correction was not employed to avoid potential loss of false-negative results.

### Western blot assay

Total protein was extracted using radioimmunoprecipitation assay (RIPA) lysis buffer (Beyotime) and detected by Western blot assays as described previously [32]. The following primary antibodies were used: MYL3 (catalog no. 66286-1; rabbit, diluted 1:1,000) and glyceraldehyde 3-phosphate dehydrogenase (GAPDH; catalog no. 60004-1; mouse, diluted 1:3,000) (both Proteintech, Chicago, IL, USA); phospho (p)-PI3K p85 (Tyr^458^)/p55 (Tyr^199^) (catalog no. 4228; rabbit, diluted 1:1,000), PI3K p110α (catalog no. 4249; rabbit, diluted 1:1,000), p-mTOR (Ser^2448^) (catalog no. 5536; rabbit, diluted 1:1,000), mTOR (catalog no. 2983; rabbit, diluted 1:1,000), p-p44/42 MAPK (ERK1/2) (Thr^202^/Tyr^204^) (catalog no. 4370; rabbit, diluted 1:1,000), p44/42 MAPK (ERK1/2) (catalog no. 4695; rabbit, diluted 1:1,000), p-Akt (Ser^473^) (catalog no. 4060; rabbit, diluted 1:1,000), Akt (catalog no. 4691; rabbit, diluted 1:1,000), Beclin-1 (catalog no. 3495; rabbit, diluted 1:1,000), and LC3B (catalog no. 2775; rabbit, diluted 1:1,000) (all Cell Signaling Technology, Beverly, MA, USA). Protein levels were determined using Image Lab produced by Bio-Rad (Waukesha, WI, USA). The quantitative analysis of blots was accomplished by using ImageJ software. GAPDH was used as an internal control for total proteins.

### RNA isolation and qRT-PCR

Nuclear and cytoplasmic RNAs were extracted from Ang II-induced NMCMs using a Cytoplasmic & Nuclear RNA Purification Kit (Norgen Biotek, Canada). The RNA concentration was determined, and the total amount of nuclear and cytoplasmic RNA was calculated for qRT-PCR.

Total RNA was isolated from NMCMs or hearts as described below. Briefly, RNA samples treated with DNase I (Takara, Japan) were quantified using a NanoDrop spectrophotometer and then reverse transcription was performed by utilizing HiScript III RT SuperMix for qPCR (+ gDNA wiper; Vazyme, Nanjing, China). Subsequently, the ChamQ Universal SYBR qPCR Master Mix (Vazyme) was applied to achieve the amplification step on the Applied Biosystems QuantStudio 3 (Thermo Fisher Scientific, Waltham, MA, USA).

The circRNA sequences were downloaded from National Center for Biotechnology Information (NCBI), and divergent PCR primers were devised based on the circRNA junction sites, with primers evenly distributed on both sides of the junction to ensure that the PCR product covered the junction site. According to a relative standard curve, each transcript’s relative mRNA level was normalized to the reference genes. The detailed primer sequences for specific genes are summarized in Table S3.

### RNase R

The circRNA characteristics were confirmed using RNase R. NMCMs were employed to extract RNAs, which were divided into 2 equal amounts for RNase R and Mock samples. Briefly, total RNA (2 mg) samples were treated with RNase R (3 U/μg) for 30 min at 37 °C, and qRT-PCR was conducted to determine the expression levels of circ-0001283.

### Virus infection and siRNA transfection

Lentivirus shcirc-0001283 and small interfering RNAs (siRNAs) for MYL3 and CSRP3 were obtained from GenePharma (Shanghai, China). The sequences are summarized in Table S4. NMCMs were cultured in 6-well plates at 1 × 10^6^ cells per well and then infected with virus or transfected with siRNA using Lipofectamine 3000 for 48 h after stimulation with or without Ang II for 24 h.

### Measurement of cell surface area

Cell surface area measurement was performed by acquiring images on a confocal laser microscope (LSM 800, Zeiss, Oberkochen, BW, Germany). NMCMs grown in confocal dishes were immobilized using 4% formaldehyde, following treatment with PBS containing 0.1%Triton X-100 for permeabilization. Next, cells were treated with α-actinin antibodies (Abcam, Carlsbad, CA, USA) overnight, followed by the addition of anti-RFP (red fluorescent protein) antibodies (Abcam) for 45 min, and incubation with 4′,6-diamidino-2-phenylindole for 10 min at room temperature. Image-Pro Plus software was applied to analyze the relative cell size by randomly selecting 50 to 100 cardiomyocytes from each group.

For immunofluorescence staining of LC3B in NMCMs, the cells were treated as for α-actinin staining. Briefly, cells were treated with anti-LC3B antibodies (Cell Signaling Technology) at 4 °C for 12 h, subsequently staining with anti-rabbit GFP (green fluorescent protein) antibodies (Abcam). A confocal laser microscope (LSM 800, Zeiss) was applied to obtain the images.

### RIP assay

RIP was carried out to verify RNA–protein interactions using a RIP Kit (Millipore, Boston, MA, USA). Briefly, the cell pellet was resuspended in RIP lysis buffer supplemented with protease inhibitor and RNase inhibitor. The NMCM lysate was treated with antibodies against MYL3 and IgG control (Proteintech) for 12 h at 4 °C. The RNA–protein complexes were then incubated with proteinase K buffer and measured by qRT-PCR.

### RNA pull-down assay

RNA pull-down was executed using the Pierce Magnetic RNA-Protein Pull-Down Kit (Thermo Fisher Scientific, Waltham, MA, USA). For circRNA pulldown, the positive probe sequence was designed to specifically recognize the back-splicing sequence of the circRNA. The sequence of negative control (NC) probe was reverse-complementary to the positive probe. The probes were synthesized with a biotinylated modification and a short space sequence of 3 adenines at their 5′ ends. In vitro transcribed circ-0001283 probe (5′ Biotion-aaaATCCATTGGACTGGATGAAGGCAAGCTGGAGG 3′) or NC probe (5′ Biotion-aaaCCTCCAGCTTGCCTTCATCCAGTCCAATGGAT 3′) for pull-down assays were obtained utilizing an AmpliScribe T7 High Yield Transcription Kit (Epicentre, Madison, WI, USA). Specifically, 10^7^ cells were washed with precooled PBS. The collected cell pellets were transferred to immunoprecipitation lysis buffer (1 ml) for lysis, following that the lysate was centrifuged at 12,000*g* for 15 min at 4 °C to separate the supernatant. Washed streptavidin-coated magnetic beads (50 μl) were incubated with biotinylated circ-0001283 (5 μg) or NC (5 μg) for 30 min at room temperature with sustained shaking. Probe-coated beads were then mixed with the cell lysis supernatant (500 μl) for 1 h. Subsequently, the beads were briefly washed 5 times with a washing buffer and eluted. Purification of the bound proteins and RNAs was performed on the pull-down materials for further analysis. Purified circ-0001283 (circ-0001283^+^) and NC (circ-0001283^-^) pull-down materials were detected using liquid chromatography/tandem mass spectrometry by Cloudseq (Shanghai, China).

### IF-FISH

Protein-RNA labeling was carried out using a Fluorescence in Situ Hybridization Kit for RNA (Beyotime). Specifically, NMCMs or hypertrophic NMCMs were incubated with hybridization solution containing circ-0001283-probe (5′-CY3-CATTGGAC+TGGA+TGAAGGCAAGC+TGGAGGGC-3′) for 5 h at 60 °C and then stained with MYL3 antibody, followed by incubation with anti-rabbit GFP antibodies (Abcam). Images were captured on a confocal laser microscope (LSM 800, Zeiss).

### CHX-chase assay

CHX (Selleck Chemicals, Houston, TX, USA) is a protein synthesis inhibitor. Cells were treated with CHX (20 μg/ml) after infection with shcirc-0001283 or shNC virus for 48 h. MYL3 protein expression was then determined by Western blot assay at 0, 0.5, 1, 2, and 4 h.

### Ubiquitination assay

NMCMs were transfected with hemagglutinin-labeled ubiquitin (HA-Ub) vector and shMYL3 virus with or without shcirc-0001283 virus for 48 h and then incubated with the proteasome inhibitor MG-132 (20 μM) (Selleck Chemicals) for 2 h. The lysates were acquired from cells by using RIPA lysis buffer (Beyotime) and then incubated with MYL3 antibody and Protein A/G beads (Life Technologies, Carlsbad, CA, USA) overnight at 4 °C for immunoprecipitation. Finally, the proteins were detected by Western blot assay.

### Statistics

All data were presented as the mean ± SEM. Differences between 2 groups were analyzed by Student’s *t* tests, and differences among 3 or more groups were analyzed by 1-way or 2-way analysis of variance (ANOVA). *P* values < 0.05 were considered statistically significant. Each experiment was repeated independently at least 3 times, with similar results obtained on each occasion.

## Data Availability

The data that support the findings of this study are available from the corresponding author upon reasonable request.
