# Supplementary material for: Circ-0001283 Aggravates Cardiac Hypertrophy by Targeting Myosin Light Chain 3 Protein
Source: Research (Wash D C). 2025 Feb 25;8:0626. doi: 10.34133/research.0626 (PMC11850654; doi:10.34133/research.0626)
Supplement: Supplementary 1 — Figs. S1 to S3 Tables S1 to S4 [file research.0626.f1.docx]

**Supplementary materials**


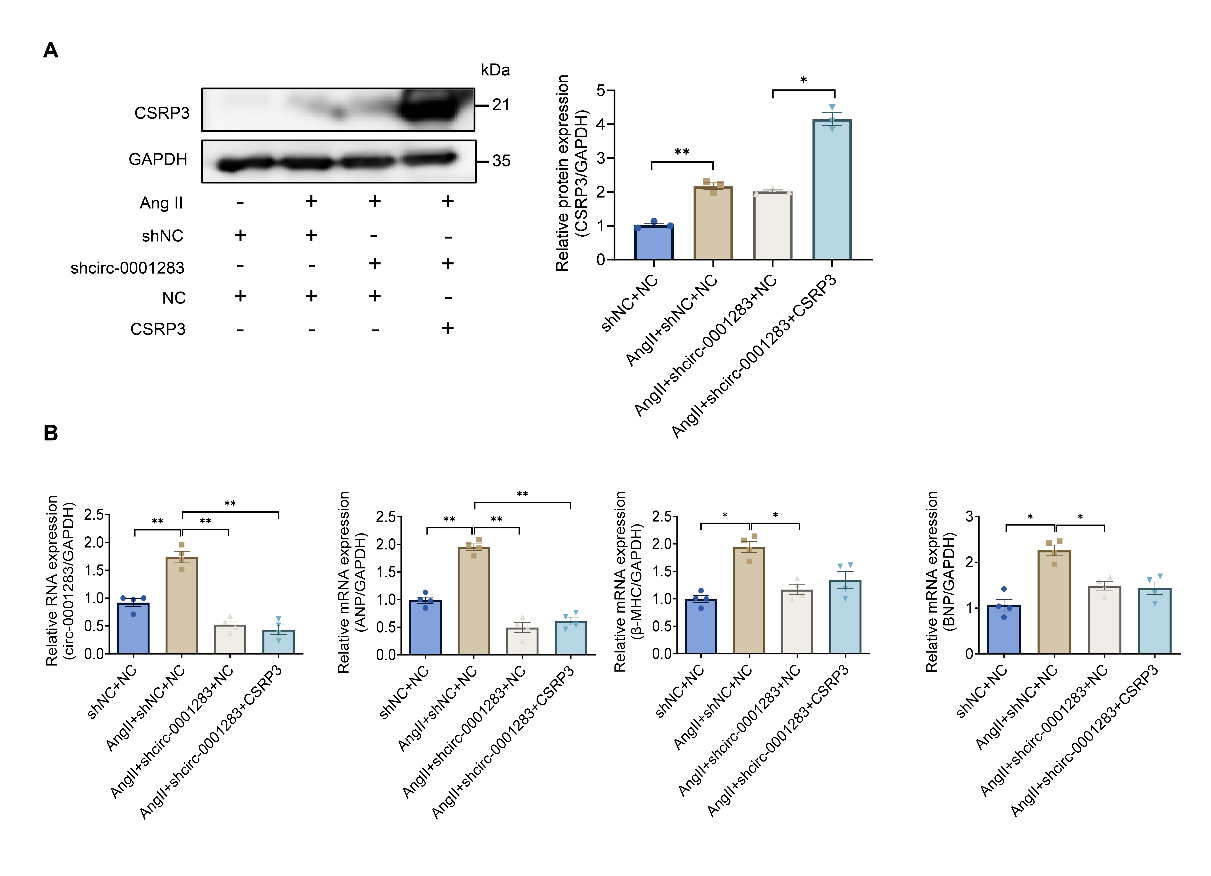


**Figure S1. Circ-0001283 regulated NMCM hypertrophy, not via CSRP3.** (**A**) Western blot analysis of CSRP3 expression in NMCMs exposed to Ang II (1 μg/mL) and transfected with shcirc-0001283 and/or CSRP3. (**B**) qRT-PCR analysis of circ-0001283, ANP, β-MHC, and BNP expression in NMCMs exposed to Ang II (1 μg/mL) and transfected with shcirc-0001283 and/or CSRP3. *p<0.05, **p<0.01, n = 3.


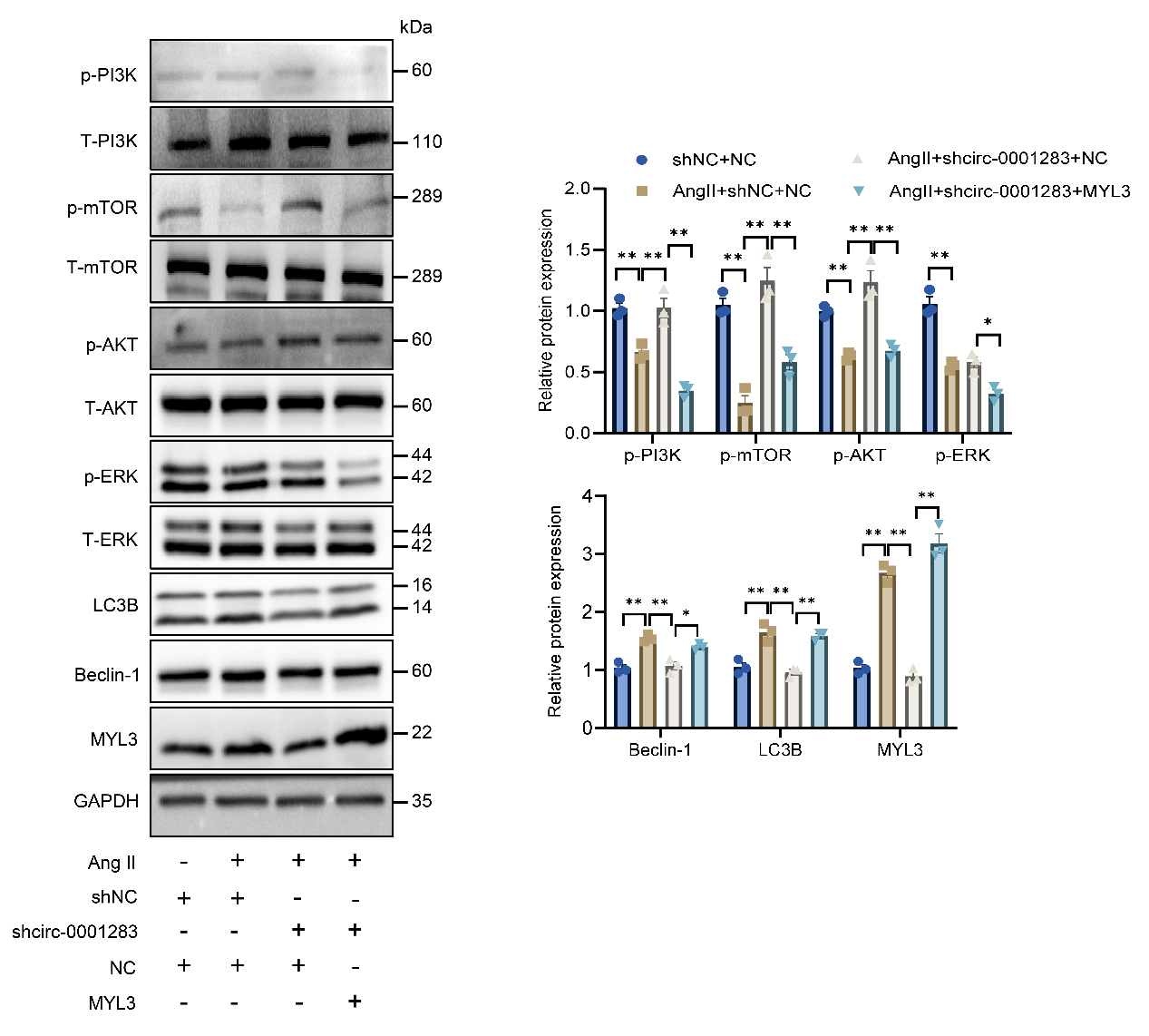


**Figure S2. Circ-0001283 regulated PI3K/Akt/mTOR and ERK pathways in NMCMs.** Western blot showing expression of p-PI3K, p-mTOR, p-Akt, p-ERK, Beclin-1, LC3B, and MYL3 in NMCMs transfected with shcirc-0001283 or MYL3 with or without Ang II (1 μg/mL). *p<0.05, **p<0.01, n = 3.


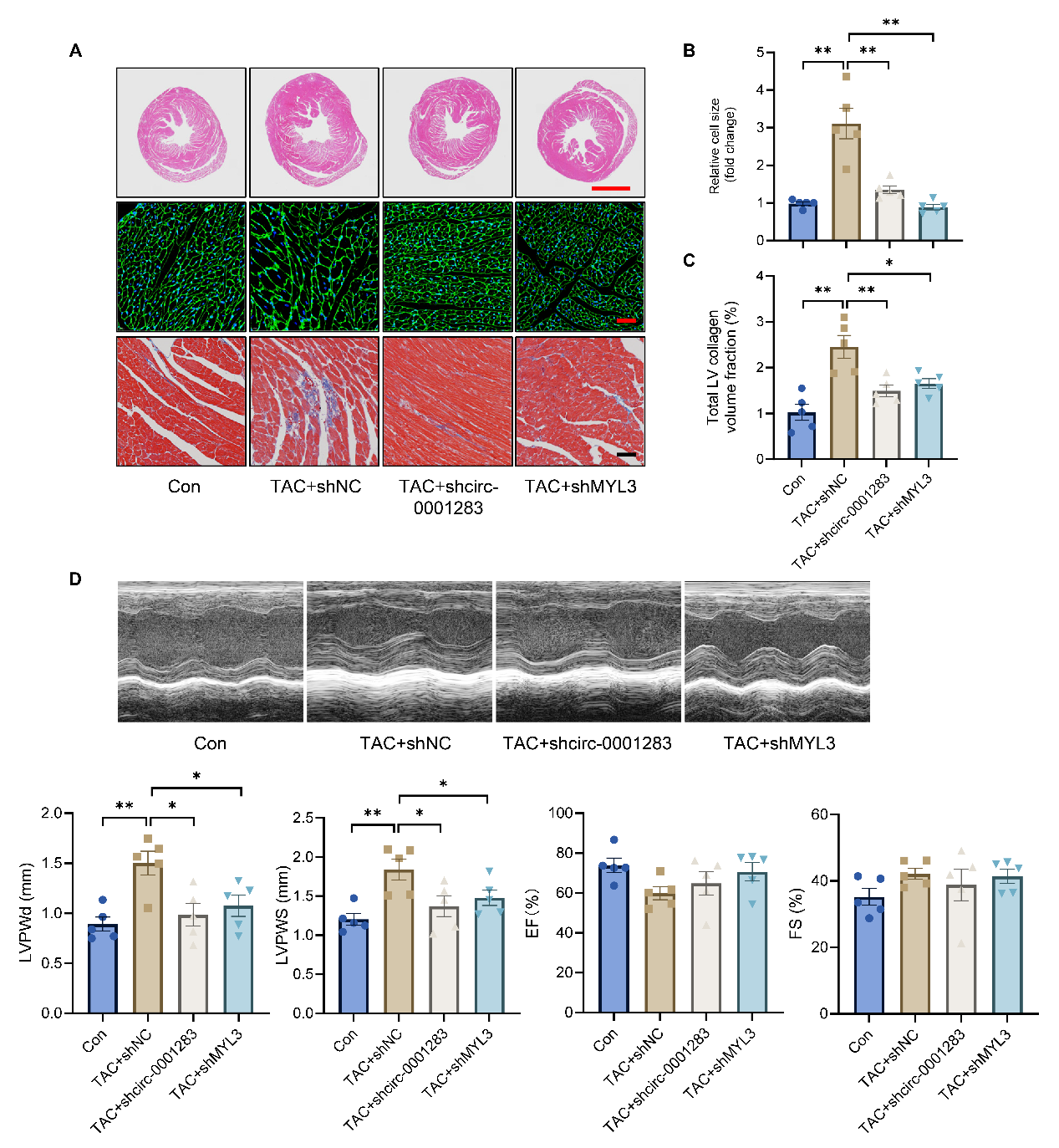


**Figure S3. Circ-0001283 or MYL3 knockdown alone reduced cardiac hypertrophy in mice.** Mice were injected with AAV9-shcirc-0001283 or AAV9-shMY3 (1 × 10^11^ vector genomes per mouse) via intravenous injection to knockdown expression of circ-0001283 or MYL3. (**A**) Representative hematoxylin and eosin (H&E), wheat germ agglutinin (WGA), and Masson’s-trichrome-stained transverse sections of hearts. Scale bar, 2 mm, 200μm, 200μm. (**B**) Quantitative analysis of WGA-stained left ventricular muscle sections. (**C**) Quantitative analysis of Masson’s-trichrome-stained histological sections. (**D**) Upper: Representative echocardiography images of mice 4w after TAC. Lower: Left ventricular posterior wall thickness at end-diastole (LVPWd), left ventricular posterior wall thickness at end-systole (LVPWs), ejection fraction (EF), and fractional shorting (FS) were measured by echocardiography 4 weeks after TAC. *p<0.05, **p<0.01, n = 5.

**Table S1. circ RNA conservation Mouse to Human**

| **Mouse circRNA (MM10)** | **human circRNA (HG19)** | | | | | |
| --- | --- | --- | --- | --- | --- | --- |
| **circRNA** | **Human_circRNA** | **original_ID** | **chrom** | **txStart** | **txEnd** | **strand** |
| chr8:80800101-80800395- | hsa-circ-0125421 | hsa-circ-0125421 | chr4 | 144336629 | 144336924 | + |
| chr4:150406196-150406664+ | hsa-circ-0114356 | hsa-circ-0114356 | chr1 | 8716031 | 8716500 | - |
| chr11:65218529-65233184- | hsa-circ-0042103 | hsa-circ-0042103 | chr17 | 12608444 | 12626325 | + |
| chr4:133719537-133723051- | hsa-circ-0008494 | hsa-circ-0008494 | chr1 | 27056141 | 27059283 | + |
| chr12:51772274-51772705- | hsa-circ-0031485 | hsa-circ-0031485 | chr14 | 31602443 | 31602881 | - |
| chr5:23979488-23991837- | hsa-circ-0006307 | hsa-circ-0006307 | chr7 | 22999874 | 23023664 | - |
| chr7:134874058-134876315+ | hsa-circ-0002456 | hsa-circ-0002456 | chr10 | 128923737 | 128926028 | + |
| chr2:37624173-37647285- | hsa-circ-0088421 | hsa-circ-0088421 | chr9 | 125920290 | 125946577 | - |
| chr1:53256629-53282092- | hsa-circ-0001083 | hsa-circ-0001083 | chr2 | 190656515 | 190682906 | + |

**Table S2. Sequence of circ-0001283**

| **>chr4:133719537-133723051-** |
| --- |
| TCATCCAGTCCAATGGATCAGATGGGAAAGATGAGACCTCAGCCGTATGGTGGGACTAACCCATACTCGCAACAACAGGGACCTCCTTCAGGACCGCAACAAGGACATGGGTACCCAGGGCAGCCATATGGGTCCCAGACTCCACAGCGGTACCCCATGACCATGCAGGGCCGGGCTCAGAGTGCCATGGGCAGCCTCTCTTATGCACAGCAGATTCCACCTTATGGCCAGCAAGGCCCCAGTGCGTATGGCCAGCAGGGCCAGACTCCATACTATAACCAGCAAAGTCCTCATCCCCAGCAGCAGCCACCTTACGCCCAGCAACCACCATCCCAGACCCCTCATGCCCAGCCTTCGTATCAGCAGCAGCCGCAGACTCAGCAACCACAGCTTCAGTCCTCTCAGCCTCCATATTCCCAGCAGCCATCCCAGCCTCCACATCAGCAGTCCCCAACTCCATATCCCTCCCAGCAGTCCACCACACAACAGCATCCCCAGAGCCAGCCCCCCTACTCACAACCACAGGCACAGTCTCCCTACCAGCAGCAGCAACCTCAGCAGCCAGCATCCTCGTCGCTCTCCCAGCAGGCTGCATATCCTCAGCCCCAGCCTCAGCAGTCCCAGCAAACTGCCTATTCCCAGCAGCGCTTCCCTCCACCACAGGAGCTTTCTCAAGATTCATTTGGGTCTCAGGCATCCTCAGCCCCCTCAATGACCTCCAGTAAGGGAGGGCAAGAAGATATGAACCTGAGTCTTCAGTCAAGGCCCTCCAGCTTGCCT |

**Table S3. Primer sequences of genes for RT-qPCR**

| **Primers** | **Forward** | **Reverse** |
| --- | --- | --- |
| circ-0001283 | CCCCTCAATGACCTCCAGTA | GGGTACCCATGTCCTTGTTG |
| ANP | CCAGCATGGGCTCCTTCTCCA | CCGGAAGCTGTTGCAGCCTAGT |
| β-MHC | CCGAGTCCCAGGTCAACAA | CTTCACGGGCACCCTTGGA |
| BNP | TAGCCAGTCTCCAGAGCAATTC | TTGGTCCTTCAAGAGCTGTCTC |
| MYL3 | TCACACCTGAACAGATTGAAGA | CCCGTATGTGATCTTCATCTCG |
| CSRP3 | AAAATGTGGAGCCTGTGAAAAG | GAAACAGGTCTTGTGGAAACTC |
| GAPDH | AAGGTCATCCCAGAGCTGAA | CTGCTTCACCACCTTCTTGA |

**Table S4. Sequences of virus**

| shcirc-0001283 | 5’- CTTGCCTTCATCCAGTCCAAT-3’ |
| --- | --- |
| shMYL3 | 5’- GATGCCTCCAAGATTAAGA -3’ |
| shCSRP3 | 5’- GCCUGGAGUCUACAAAUGU-3’ |
| MYL3 | 5’-ATGGCCCCCAAAAAGCCAGAGCCCAAGAAGGATGATGCCAAAGCGGC  TGCCCCCAAAGCAGCTCCAGCTCCTGCGGCTGCACCTGCAGCTGCACCTGCGGCTGCACCAGAGCCTGAGCGCCCCAAGGAAGCCGAGTTTGATGCCTCCAAGATTAAGATCGAGTTCACACCTGAACAGATTGAAGAGTTCAAGGAGGCCTTCCTGCTGTTCGACCGCACACCCAAGGGCGAGATGAAGATCACATACGGGCAGTGTGGGGATGTCCTGCGGGCTCTGGGTCAGAATCCTACCCAGGCAGAGGTGCTCCGTGTCCTGGGGAAGCCAAAACAGGAAGAGCTCAATTCCAAGATGATGGATTTTGAGACGTTCCTGCCTATGCTCCAGCACATCTCCAAGAACAAGGACACTGGCACGTACGAGGACTTCGTGGAGGGGCTGCGGGTCTTCGACAAGGAGGGCAACGGCACGGTCATGGGTGCAGAGCTCCGACACGTGCTGGCCACGCTGGGTGAGAGACTGACAGAAGATGAGGTAGAGAAACTGATGGCTGGTCAAGAGGACTCCAACGGCTGCATCAACTATGAAGCGTTTGTGAAGCATATCATGGCGAGCTGA-3’ |
| CSRP3 | 5’-ATGCCAAACTGGGGTGGAGGTGCAAAATGTGGAGCCTGTGAAAAGAC  GGTCTACCATGCAGAAGAAATCCAGTGCAATGGGAGGAGTTTCCACAAGACCTGTTTCCACTGCATGGCCTGCAGGAAAGCTCTGGACAGCACCACAGTGGCAGCTCATGAGTCAGAGATCTACTGTAAGGTGTGCTATGGGCGCAGGTATGGCCCCAAGGGGATCGGGTTCGGACAAGGCGCTGGCTGCCTCAGCACAGACACTGGCGAGCATCTTGGCCTGCAGTTCCAACAATCCCCAAAGCCAGCTCGAGCAGCCACCACAAGCAACCCTTCCAAATTCTCTGCAAAGTTTGGAGAATCAGAGAAGTGCCCACGATGTGGAAAGTCGGTATACGCTGCTGAGAAGGTCATGGGAGGTGGCAAGCCCTGGCACAAGACCTGCTTCCGCTGTGCCATCTGTGGGAAGAGCCTGGAGTCTACAAATGTCACTGACAAGGATGGGGAGCTCTACTGCAAAGTTTGCTATGCCAAAAATTTTGGCCCCACAGGCATTGGGTTTGGAGGGCTTACACAGCAAGTGGAAAAGAAGGAGTGA-3’ |
